# Knowledge, Attitudes and Practices of Physicians Regarding Antifungal Therapy in Tertiary Care Patients: A Cross-Sectional Survey in Greece

**DOI:** 10.3390/pathogens15020138

**Published:** 2026-01-26

**Authors:** Georgios Kariniotakis, Evangelos I. Kritsotakis, Stamatis Karakonstantis, Petros Ioannou, Diamantis P. Kofteridis

**Affiliations:** 1 Division of Internal Medicine, School of Medicine, University of Crete, 71003 Heraklion, Greece; medp2012173@med.uoc.gr (G.K.); stamkar2003@gmail.com (S.K.);; 2 Laboratory of Biostatistics, School of Medicine, University of Crete, 71003 Heraklion, Greece; e.kritsotakis@uoc.gr

**Keywords:** antifungal, resistance, attitudes, stewardship

## Abstract

The rising incidence of invasive fungal infections (IFIs) and the associated antifungal resistance underscore the need for antifungal stewardship (AFS) programs. Evaluating physicians’ knowledge and practices is crucial for identifying gaps and planning effective AFS interventions. A self-administered questionnaire was distributed to staff and resident physicians at a referral university-affiliated hospital in Greece in November 2025. The survey examined participants’ knowledge on fungal diagnosis and treatment, their prescribing attitudes and practices, and their AFS-related education, knowledge and preferences. In total, 70 physicians (46 residents and 24 staff consultants) participated in the survey from medical departments (63%), surgical departments (30%), and intensive care units (7%). Physicians surveyed demonstrated a low average knowledge score of 36.6% correct answers (SD, 22.7%; range 0% to 90%) regarding IFIs and antifungal agents, and significant variation was observed across different hospital departments. Awareness of risk factors for IFI varied widely, with recognition rates of different factors ranging from 10% to 100% across departments. Many physicians (71%) reported a lack of confidence in prescribing antifungal therapy and reliance on infectious disease experts was common (84%). Most preferred training methods were case-based discussions and printed guidelines. The substantial knowledge gaps and low confidence in prescribing antifungals among physicians highlight the urgent need for education and implementation of local guidelines to optimize antifungal use that might improve patient outcomes.

## 1. Introduction

Over the past two decades, the incidence of invasive fungal infections (IFIs) has escalated, leading to an estimated 3 million cases and 1.6 million related deaths annually worldwide [1]. This surge is attributable primarily to expanding populations of immunocompromised patients, including those receiving advanced therapies for malignancies, autoimmune diseases, and organ transplants, as well as elderly patients with multiple comorbidities [2]. Delayed diagnosis and suboptimal antifungal treatment contribute significantly to poor clinical outcomes [3], which emphasizes the need for rational antifungal use to mitigate toxicity, unnecessarily prolonged administration, resistance emergence, and mortality, while also curbing healthcare costs [4,5]. Antifungal resistance is a growing concern and poses significant threats to public health [4,6]. 

In Greece, invasive fungal infections (IFIs) predominantly include candidemia and invasive candidiasis, primarily caused by *Candida albicans* [7] (though non-*albicans* species such as *C. parapsilosis*, *C. glabrata*, and *C. tropicalis* are increasingly prevalent, accounting for a species shift in recent epidemiological trends) [8]. Invasive aspergillosis, mainly due to *Aspergillus fumigatus*, is also common, especially among immunocompromised patients like those with hematological malignancies or post-transplant. Less frequent but notable IFIs include mucormycosis (etiologic agents: *Rhizopus* spp., *Mucor* spp.) and cryptococcosis (*Cryptococcus neoformans*), often seen in ICU or HIV-positive populations [9]. The antifungal agents used for these infections based on the guidelines of the Infectious Diseases Society of America (IDSA), include echinocandins as first-line for invasive candidiasis; voriconazole or isavuconazole for invasive aspergillosis; liposomal amphotericin B for mucormycosis; and fluconazole or amphotericin B for cryptococcosis, with adjustments for susceptibility testing and patient comorbidities

Antifungal stewardship (AFS) programs aim to promote appropriate, timely therapy to improve patient outcomes, reduce resistance, minimize adverse effects, and lower healthcare expenses [4,5]. However, comprehensive implementations of AFS programs remain limited, particularly in Greece, where prior studies have been small and focused on specific populations [10,11]. Previous studies conducted in Greece have shown that approximately 25% of antifungal prescriptions lack a clear clinical indication, highlighting the widespread prevalence of inappropriate antifungal use [10,11].

This study aimed to evaluate knowledge, attitudes, and practices regarding antifungal therapy at a tertiary care hospital in Greece, as a pre-intervention step toward developing an effective AFS program. By identifying knowledge and practice gaps, we can guide education and interventions to promote the rational use of antifungals.

## 2. Materials and Methods

### 2.1. Study Design and Participants

A cross-sectional survey was conducted in November 2025 at the University Hospital of Heraklion, which is a 750-bed tertiary care center that admits more than 55,000 inpatients annually and serves as the referral hospital for the island of Crete, Greece. The survey targeted attending physicians and resident trainees across all hospital departments providing adult patient care (except pediatrics), and 150 self-administered questionnaires were distributed. Survey participation was voluntary and anonymous, and participants were given 10 min to complete the questionnaire.

### 2.2. Questionnaire

The survey questionnaire was developed after consulting the European survey conducted by the Collaboration in Mycology Study Group [12], with modifications and additional questions guided by infectious disease experts. The complete questionnaire is presented as Supplementary Material and comprises five sections:

- *Section 1 “Demographics”* consisted of questions about specialty, years of practice, and position (resident or attending physician).
- *Section 2 “Knowledge of IFIs and antifungal agents”* contained 10 questions on treatment choice, duration, monitoring, and diagnostics. Each question included four options and an additional “I am not familiar” option.
- *Section 3 “Awareness of risk factors for IFIs”* was a multiple-choice question requesting to select the risk factors for IFIs from a list of 12 choices.
- *Section 4 “Practices on antifungals”* contained 12 questions regarding prescribing basis, confidence, consultation frequency, de-escalation, level of familiarity with different antifungal uses (prophylactic, empirical, pre-emptive, and targeted), biomarker use, and AFS confidence.
- *Section 5 “AFS knowledge and training Interests”* comprised two multiple-choice questions on AFS goals, strategies, training areas, and preferred methods (multiple allowed).

The correct answers to the questionnaire’s knowledge sections were based on the guidelines of the Infectious Diseases Society of America (IDSA).

### 2.3. Statistical Analysis

The knowledge-testing questions in Section 2 of the questionnaire were coded as correct or false, with “I am not familiar” responses included in the false category for analysis as it reflects a lack of actionable knowledge for clinical decision-making.

An additive performance score (0–100) was assigned to each physician, reflecting the percentage of correct answers in Section 2 (random guessing would have yielded a mean of 20% correct answers). Mean differences in knowledge scores between different physician groups (classified by specialty, type of post, and years of clinical practice) were estimated using linear regression. Frequency distributions in the responses to single questions were compared across different groups of physicians using Pearson’s chi-square test or Fisher’s exact test as appropriate. 

### 2.4. Ethics

The study was approved by the Institutional Ethics Review Board of the University Hospital of Heraklion on 14 March 2025 (approval reference number 46/7.3.2025).

## 3. Results

### 3.1. Participants

The University Hospital of Heraklion is a major tertiary referral center in Greece, with approximately 750 beds. It provides comprehensive secondary and tertiary care across a wide range of specialties. The hospital serves as the primary referral facility for the island of Crete and nearby islands, covering a population of about 650,000 residents. A total of 70 physicians participated in the study, of whom 44 (63%) were in medical departments (29 residents and 15 staff), 21 (30%) were in surgical departments (14 residents, 7 staff), and 5 (7%) were in the adult intensive care unit (ICU) (3 residents, 2 staff). 

### 3.2. Knowledge of IFIs and Antifungal Agents

Physicians surveyed demonstrated a low average knowledge score of 36.6% correct answers (SD, 22.7%; range 0% to 90%) regarding IFIs and antifungal agents. Staff physicians, physicians with 11–20 years of clinical experience, and those working in medical departments and the ICU achieved the highest knowledge scores (Table 1). Regarding years of clinical practice, univariate analysis indicated that physicians with 11–20 years of experience achieved the highest knowledge scores (52.5%), potentially reflecting accumulated exposure to IFIs through routine patient management and professional development. However, this association was not significant in multivariable regression (adjusted *p* > 0.05), likely confounded by department specialty, as more experienced staff are often concentrated in medical or ICU settings where IFI encounters are more frequent. This suggests that while seniority may enhance knowledge through practical experience, targeted training is essential across all experience levels to address gaps, particularly in less exposed groups like early-career physicians. Small sample sizes in certain experience categories (e.g., *n* = 8 for 11–20 years) may have limited statistical power, warranting larger studies to explore this further.

Multivariable regression analysis showed that only the department specialty was independently and significantly associated with the knowledge scores. Physicians working in surgical departments had significantly lower scores than those working in medical departments (mean difference: −21%; 95% CI: −32% to −10% in percent correct responses; *p* < 0.001). The distributions of correct responses to individual knowledge-testing questions are shown in Table 2 for the entire respondent pool and by the clinical department. Overall, the highest performance was observed for knowledge about candidemia treatment duration (66% correct responses), factor necessitating dosing adjustment (56% correct), and aspergillosis therapy (53% correct), but physicians from the surgical departments performed lower on those questions. Gastrointestinal rupture prophylaxis (4% correct) and candida colonization index threshold (7% correct) were the least known aspects of IFI management among the surveyed physicians.

### 3.3. Risk Factors for IFIs

In the risk factors for IFIs section (Table 3), physicians demonstrated varying levels of awareness, with recognition rates of different factors ranging from 10% to 100% across departments. Common risk factors, such as corticosteroid use, diabetes mellitus, and immunosuppression, were recognized at relatively high overall frequencies (83%, 79%, and 76%, respectively), reflecting general familiarity with well-established predisposing conditions. However, surgeons were less aware of these common risk factors, and the respective awareness percentages were statistically significantly lower in surgical departments (67%, 67%, and 52%, respectively). Awareness of less frequently highlighted factors, such as allogeneic hematopoietic stem cell transplantation (Allo-HSCT) and multifocal *Candida* spp. colonization, was notably lower, with awareness percentages ranging from 14% to 80% and 29% to 60%, respectively, across specialties. Abdominal surgery was the least recognized risk factor for IFI (overall, 33% awareness), even among the surgeons (10% awareness).

### 3.4. Physicians’ Practices

The third part of the questionnaire provided extensive insights into the physicians’ practices, knowledge, and confidence in antifungal therapy. The results are summarized in Table 4. Antifungal therapy practices revealed inconsistencies in consulting infectious disease experts, with up to 40% in the ICU and 9% in the medical wards rarely or never seeking expert opinions. A significant number of physicians (80%) do not routinely review cases to de-escalate antifungal therapy. Knowledge deficits were apparent. In various departments, up to 80% of physicians were unfamiliar with prophylactic use, up to 81% unfamiliar with pre-emptive use, and up to 100% unfamiliar with diagnostic tools like galactomannan and beta-D-glucan; consequently, 60% to 90% reported not being confident in prescribing according to antifungal stewardship principles. Prophylactic refers to preventive antifungal use in high-risk patients without infection (e.g., neutropenia); empirical means initiating therapy based on clinical suspicion without confirmed diagnosis (e.g., persistent fever); pre-emptive involves starting treatment based on biomarkers or risk factors indicating early infection, before full confirmation [12].

### 3.5. AFS Knowledge and Training

This section revealed knowledge gaps among physicians. The results are summarized in Table 5. About 38% of respondents correctly identified the primary goal of AFS as optimizing use, improving outcomes, and reducing resistance. Just 29% selected the guideline-aligned initial strategy for suspected candidiasis, which is a diagnostic tools-based treatment initiation. Physicians demonstrated strong demand for additional training, specifically in diagnostic tools (56%), de-escalation principles (37%), and resistance management (33%). When asked about preferred educational methods, physicians favored practical approaches, with case-based discussions and printed guidelines selected by 49% and 46% of the respondents, respectively.

## 4. Discussion

This survey provides a detailed assessment of physicians’ knowledge, attitudes, and practices regarding the management of invasive fungal infections in a large tertiary-care hospital in Greece. The findings reveal substantial knowledge gaps and practice variability across specialties, underscoring the urgent need for structured antifungal stewardship initiatives.

Overall knowledge of antifungal therapy and IFI management was low, with an average accuracy rate far below what would be expected for safe and effective clinical decision-making. The wide score range highlights significant heterogeneity within the physician workforce. Specialty emerged as the only independent predictor of performance, with physicians in surgical departments displaying consistently poorer knowledge. The exceptionally low recognition of topics such as colonization thresholds and prophylaxis strategies suggests limited exposure to mycology-specific training during routine clinical practice.

Awareness of risk factors for invasive fungal disease demonstrated a similar pattern. While common predisposing conditions were generally well recognized, surgical departments showed markedly lower awareness even of widely acknowledged risks. The striking under-recognition of abdominal surgery as a major risk factor is clinically significant, given the high burden of *Candida* spp. infections in postoperative intra-abdominal settings [13]. This gap may contribute to delays in diagnosis, suboptimal empiric choices, and inconsistent use of risk-driven diagnostic algorithms. Additionally, the low awareness of high-risk immunologic states, such as allogeneic stem cell transplantation, may reflect limited interdisciplinary communication and insufficient interaction with hematology-oncology services.

Reported clinical practices further reinforce concerns regarding the quality of antifungal utilization. Many physicians infrequently consult infectious disease specialists, and most do not routinely review de-escalation therapies, a cornerstone of stewardship. The near-universal unfamiliarity with key diagnostic biomarkers such as galactomannan and β-D-glucan is particularly concerning, as these tools are essential to avoiding unnecessary empirical therapy and improving diagnostic precision [14]. A limited understanding of prophylactic, empirical, and preemptive strategies further suggests that prescribing decisions may rely heavily on individual judgment rather than standardized protocols. Collectively, these findings likely contribute to the high rates of inappropriate antifungal use previously documented in Greece [10].

The observed deficiencies in antifungal stewardship knowledge highlight the absence of comprehensive, hospital-wide stewardship structures. Less than half of the participants correctly identified the fundamental goals of stewardship programs, and only a minority demonstrated awareness of guideline-based approaches to early management of suspected candidiasis. At the same time, the strong interest in further training, particularly in diagnostic modalities, de-escalation strategies, and resistance mechanisms, indicates an environment receptive to targeted interventions.

Taken together, these results point to several priorities for developing a local antifungal stewardship program. First, educational interventions should be tailored to specialties with consistently lower levels of knowledge, particularly surgical services. Second, structured training should emphasize risk assessment, biomarker interpretation, and clear indications for initiating, continuing, or de-escalating therapy. Third, multidisciplinary collaboration, especially between surgeons, intensivists, hematologists, and infectious disease experts, should be promoted to support timely and accurate decision-making. Finally, the creation of locally adapted guidelines and case-based teaching sessions may be particularly effective, given the preferences expressed by the surveyed physicians.

This study aligns with prior European research, particularly the 2015 multicenter survey by Valerio et al. [15], which assessed knowledge of invasive fungal infections (IFIs) among 121 prescribing physicians across Spain, Italy, Denmark, and Germany. In that study, participants achieved a mean knowledge score of 5.8 out of 10 (equivalent to 58%), higher than the 36.6% observed in this study, though differences in questionnaire design (20 questions scored at 0.5 points each versus 10 questions scored at 1 point) limit direct comparability. Departmental variations are consistent: medical specialties performed better in both surveys (medical departments scored higher than ICUs or surgical departments), underscoring limited familiarity in non-infectious disease-focused areas. Practices also overlap, with the European study noting 38% awareness of prophylaxis indications and 47% correct first-line choices for unspecified IFIs, similarly to this study’s inconsistencies in de-escalation (76% not routine) and familiarity with strategies (e.g., 70% unfamiliar with pre-emptive use). Confidence was inferred as low in the European context through performance gaps, paralleling this study’s 71% overall lack of confidence and 75% reliance on infectious disease experts. No other large-scale European surveys on physicians’ knowledge were identified after 2015 directly matching this scope, though a 2020 Italian pilot study suggested ongoing doubts in understanding IFIs, with inappropriate antifungal use potentially reaching 75% in some settings [16].

Similar research in other regions highlights a global need for improved medical education regarding IFIs. In Nigeria, a multicenter survey of 1046 resident doctors across seven tertiary hospitals evaluated knowledge and awareness of IFIs, finding that while 80% had some awareness (primarily from undergraduate or postgraduate training), overall knowledge was suboptimal, with only 0.2% (2 participants) achieving a “good” level (≥70% correct responses) and the majority categorized as poor (<40%) or fair (40–69%) [17]. This underscores widespread gaps in diagnostic and management practices among trainees in resource-limited settings. In Colombia, the FungiCAP Survey involving 285 physicians (primarily from internal medicine) revealed significant deficiencies, with 87% lacking knowledge about antifungal pharmacokinetics, 61% lacking knowledge about pharmacodynamics, and 64% demonstrating poor understanding of the appropriate drug of choice by disease type; additionally, 78% felt their undergraduate education was insufficient for prescribing antifungals, emphasizing the need for enhanced curricula [18]. In Saudi Arabia, a cross-sectional study of 63 healthcare professionals in critical care settings (including ICU physicians and clinical pharmacists) showed that only 3% had good overall knowledge of systemic antifungal prescribing, with 51% rated as poor and 46% as moderate, highlighting specific gaps such as incorrect initiation of empiric therapy (e.g., only 24% correct for septic shock in dialysis patients) and uncertainty in special populations like pregnancy [19].

This study has several limitations that should be considered when interpreting its findings. First, even though the study was conducted at a setting typical of tertiary care provision in Greece, the single-center design might limit the generalizability of the results to other institutions, healthcare systems, or regions with different patient populations and antifungal prescribing practices. Second, the relatively small sample size and moderate response rate may have introduced selection bias, as physicians with greater interest or awareness of antifungal therapy may have been more likely to participate. Additionally, the uneven representation across hospital departments, particularly the underrepresentation of intensive care unit physicians, may have influenced overall knowledge estimates and limited conclusions regarding certain high-risk clinical settings. The reliance on self-administered questionnaires introduces the possibility of reporting and recall biases, including overestimation of confidence or adherence to recommended practices. Furthermore, the cross-sectional design captures practices and knowledge at a single time point, and does not allow assessment of temporal trends, causal relationships, or the impact of educational or stewardship interventions. Finally, although the questionnaire was adapted from a previously published European survey [15] and reviewed by experts, the absence of formal validation in the local context may affect the reliability and interpretability of the results.

## 5. Conclusions

This survey reveals significant gaps in physicians’ knowledge and practices regarding invasive fungal infections and antifungal therapy, particularly within surgical specialties at a major tertiary hospital in Greece. Limited familiarity with risk factors, diagnostics, and stewardship principles contributes to inconsistent and often inappropriate antifungal use. The strong interest in further training emphasizes the need for a structured antifungal stewardship program with targeted education to improve prescribing quality and patient outcomes.

## Figures and Tables

**Table 1:** Differences in mean performance scores regarding knowledge of invasive fungal infections and antifungal agents by physician characteristics.

Physician Group	n (%)	Mean ± SD	MD (95% CI)	Adjusted MD (95% CI) *	*p*
Department specialty					
Medical	44 (63%)	43.6 ± 21.9	Ref.	Ref.	
Surgical	21 (30%)	21.0 ± 16.7	−22.7 (−33.5 to −11.8)	−21.2 (−32.3 to −10.0)	<0.001
Intensive care	5 (7%)	40.0 ± 22.4	−3.6 (−23.0 to 15.7)	−2.4 (−22.0 to 17.2)	0.81
Years of clinical practice					
<5	36 (51%)	34.2 ± 17.8	−18.3 (−35.8 to −0.8)	−3.0 (−29.6 to 23.6)	0.82
5–10	16 (23%)	36.2 ± 22.2	−16.2 (−35.6 to 3.1)	−6.2 (−28.6 to 16.1)	0.58
11–20	8 (11%)	52.5 ± 31.1	Ref.	Ref.	
>20	10 (14%)	33.0 ± 29.8	−19.5 (−40.7 to 1.7)	−11.3 (−31.3 to 8.7)	0.26
Physician category					
Staff	24 (34%)	42.5 ± 30.0	Ref.	Ref.	
Resident	46 (66%)	33.5 ± 17.4	−9.0 (−20.3 to 2.3)	−11.3 (−32.5 to 9.8)	0.29

Abbreviations: SD, standard deviation; MD, mean difference; CI, confidence interval; Ref., reference category. * Adjusted mean differences were estimated by multiple linear regression accounting for all three variables simultaneously.

**Table 2:** Distribution of correct answers on knowledge questions concerning invasive fungal infections and antifungal agents by department specialty.

Question (Correct Answer)	Overall (n = 70)	Department Specialty			
		Medical (n = 44)	Surgical (n = 21)	ICU (n = 5)	*p*
Initial treatment for invasive candidiasis (*Candida* spp.) in an unstable patient (echinocandin)	32 (46%)	23 (52%)	5 (24%)	4 (80%)	0.027
Duration for uncomplicated candidemia (*Candida* spp.) after cultures have cleared (14 days)	46 (66%)	34 (77%)	9 (43%)	3 (60%)	0.023
Agent requiring drug monitoring (voriconazole)	34 (49%)	24 (55%)	8 (38%)	2 (40%)	0.43
First choice for invasive aspergillosis (*Aspergillus* spp.) (voriconazole/isavuconazole)	37 (53%)	30 (68%)	5 (24%)	2 (40%)	0.003
Factor not necessitating dosing adjustment (patient age)	39 (56%)	26 (59%)	10 (48%)	3 (60%)	0.67
Most sensitive test for early detection of aspergillosis (*Aspergillus* spp.) (serum galactomannan assay)	27 (39%)	19 (43%)	5 (24%)	3 (60%)	0.19
Component not in the Candida score (neutropenia)	8 (11%)	8 (18%)	0 (0%)	0 (0%)	0.07
Candida colonization index threshold (0.5)	5 (7%)	5 (11%)	0 (0%)	0 (0%)	0.20
Gastrointestinal rupture requiring prophylaxis against *Candida* spp. (both oesophagus and colon)	3 (4%)	2 (5%)	1 (5%)	0 (0%)	0.89
Antifungal therapy in neutropenic fever is indicated (after 72 h of empirical antibiotics)	25 (36%)	21 (48%)	1 (5%)	3 (60%)	0.002

ICU: intensive care unit.

**Table 3:** Physicians’ awareness of risk factors for invasive fungal infection by department specialty.

Risk Factor	Overall (n = 70)	Department Specialty			
		Medical (n = 44)	Surgical (n = 21)	ICU (n = 5)	*p*
Recent broad-spectrum antibiotic use	34 (49%)	24 (55%)	6 (29%)	4 (80%)	0.052
Diabetes mellitus	55 (79%)	38 (86%)	14 (67%)	3 (60%)	0.095
Prolonged and profound neutropenia	45 (64%)	34 (77%)	7 (33%)	4 (80%)	0.002
Chronic kidney disease	25 (36%)	18 (41%)	5 (24%)	2 (40%)	0.41
Hematologic malignancy	46 (66%)	35 (80%)	7 (33%)	4 (80%)	<0.001
Allo-HSCT	34 (49%)	27 (61%)	3 (14%)	4 (80%)	<0.001
Immunosuppression	53 (76%)	37 (84%)	11 (52%)	5 (100%)	0.012
Total parenteral nutrition	40 (57%)	31 (70%)	6 (29%)	3 (60%)	0.004
Abdominal surgery	23 (33%)	18 (41%)	2 (10%)	3 (60%)	0.010
Central venous catheter	38 (54%)	29 (66%)	5 (24%)	4 (80%)	0.002
Multifocal candida colonization	34 (49%)	25 (57%)	6 (29%)	3 (60%)	0.10
Corticosteroid use	58 (83%)	40 (91%)	14 (67%)	4 (80%)	0.044

**Table 4:** Physicians’ practices on antifungal medications.

Questions and Responses	Overall (n = 70)	Department Specialty			
		Medical (n = 44)	Surgical (n = 21)	ICU (n = 5)	*p*
Antifungal prescribing in your department is mostly based on:					
Local guidelines	12 (17%)	4 (9%)	4 (19%)	4 (80%)	<0.001
Stanford guide	6 (9%)	4 (9%)	1 (5%)	1 (20%)	0.539
Infectious diseases consultants	51 (73%)	37 (84%)	13 (62%)	1 (20%)	0.004
Therapy protocol	7 (10%)	6 (14%)	1 (5%)	0 (0%)	0.398
Artificial intelligence	1 (1%)	0 (0%)	1 (5%)	0 (0%)	0.306
How confident are you in interpreting antifungal susceptibility reports?					
Very	5 (7%)	3 (7%)	2 (10%)	0 (0%)	0.848
Somewhat	26 (37%)	18 (41%)	6 (29%)	2 (40%)	
Not	39 (56%)	23 (52%)	13 (62%)	3 (60%)	
How often do you consult an ID expert to treat a patient with suspected or confirmed IFI?					
Always	34 (49%)	22 (50%)	11 (52%)	1 (20%)	0.130
Often	18 (26%)	13 (30%)	5 (24%)	0 (0%)	
Sometimes	10 (14%)	5 (11%)	3 (14%)	2 (40%)	
Rarely	7 (10%)	4 (9%)	1 (5%)	2 (40%)	
Never	1 (1%)	0 (0%)	1 (5%)	0 (0%)	
How often do you prescribe antifungal medications?					
Often	11 (16%)	10 (23%)	1 (5%)	0 (0%)	0.065
Sometimes	23 (33%)	13 (30%)	6 (29%)	4 (80%)	
Rarely	22 (31%)	15 (34%)	7 (33%)	0 (0%)	
Never	14 (20%)	6 (14%)	7 (33%)	1 (20%)	
How familiar are you with the prophylactic use of antifungal medication?					
Very	8 (11%)	7 (16%)	0 (0%)	1 (20%)	0.110
Somewhat	20 (29%)	15 (34%)	5 (24%)	0 (0%)	
Not	42 (60%)	22 (50%)	16 (76%)	4 (80%)	
How familiar are you with the empirical use of antifungal medication?					
Very	6 (9%)	5 (11%)	0 (0%)	1 (20%)	0.070
Somewhat	32 (46%)	24 (55%)	7 (33%)	1 (20%)	
Not	32 (46%)	15 (34%)	14 (67%)	3 (60%)	
How familiar are you with the pre-emptive use of antifungal medication?					
Very	2 (3%)	1 (2%)	0 (0%)	1 (20%)	0.121
Somewhat	19 (27%)	14 (32%)	4 (19%)	1 (20%)	
Not	49 (70%)	29 (66%)	17 (81%)	3 (60%)	
How familiar are you with the targeted use of antifungal medication?					
Very	4 (6%)	3 (7%)	0 (0%)	1 (20%)	0.360
Somewhat	37 (53%)	25 (57%)	10 (48%)	2 (40%)	
Not	29 (41%)	16 (36%)	11 (52%)	2 (40%)	
How familiar are you with Galactomannan and B–D glucan use in clinical practice?					
Very	13 (19%)	12 (27%)	0 (0%)	1 (20%)	<0.001
Somewhat	23 (33%)	21 (48%)	0 (0%)	2 (40%)	
Not	34 (49%)	11 (25%)	21 (100%)	2 (40%)	
How often do you reassess antifungal prescriptions for de-escalation or discontinuation?					
Always	14 (20%)	13 (30%)	0 (0%)	1 (20%)	0.046
Often	8 (11%)	4 (9%)	2 (10%)	2 (40%)	
Sometimes	13 (19%)	10 (23%)	3 (14%)	0 (0%)	
Rarely	19 (27%)	9 (20%)	9 (43%)	1 (20%)	
Never	16 (23%)	8 (18%)	7 (33%)	1 (20%)	
How confident are you in prescribing antifungals according to AFS principles?					
Very	3 (4%)	2 (5%)	0 (0%)	1 (20%)	0.082
Somewhat	17 (24%)	14 (32%)	2 (10%)	1 (20%)	
Not	50 (71%)	28 (64%)	19 (90%)	3 (60%)	

**Table 5:** Areas and preferred methods for AFS training among surveyed physicians.

Questions and Responses	Overall (n = 70)	Department Specialty			
		Medical (n = 44)	Surgical (n = 21)	ICU (n = 5)	*p*
Area of IFI management or antifungal use to benefit from:					
Diagnostic tools	39 (56%)	28 (64%)	8 (38%)	3 (60%)	0.150
Principles of de-escalation	26 (37%)	18 (41%)	7 (33%)	1 (20%)	0.598
Management of resistance	23 (33%)	15 (34%)	6 (29%)	2 (40%)	0.852
Therapeutic drug monitoring	21 (30%)	15 (34%)	6 (29%)	0 (0%)	0.285
Drug-drug interactions	22 (31%)	17 (39%)	5 (24%)	0 (0%)	0.141
Preferred method for receiving AFS-related training:					
Interactive workshops	18 (26%)	12 (27%)	4 (19%)	2 (40%)	0.583
Online learning modules	14 (20%)	10 (23%)	4 (19%)	0 (0%)	0.480
Case-based discussions	34 (49%)	21 (48%)	11 (52%)	2 (40%)	0.869
Printed guidelines	32 (46%)	20 (45%)	9 (43%)	3 (60%)	0.786

## Data Availability

The database of the questionnaire can be requested via mail from the corresponding author.

## Supplementary Materials

The following supporting information can be downloaded at: https://www.mdpi.com/article/10.3390/pathogens15020138/s1.

## Abbreviations

The following abbreviations are used in this manuscript:

AFS	Antifungal Stewardship
IFI	Invasive Fungal Infection
ICU	Intensive Care Unit
IDSA	Infectious Diseases Society of America
Allo-HSCT	Allogeneic Hematopoietic Stem Cell Transplantation
SD	Standard Deviation
MD	Mean Difference
CI	Confidence Interval
Ref	Reference

## References

[1] Bongomin F., Gago S., Oladele R.O., Denning D.W. (2017). Global and Multi-National Prevalence of Fungal Diseases—Estimate Precision. J. Fungi.

[2] des Champs-Bro B., Leroy-Cotteau A., Mazingue F., Pasquier F., François N., Corm S., Lemaitre L., Poulain D., Yakoub-Agha I., Alfandari S. (2011). Invasive Fungal Infections: Epidemiology and Analysis of Antifungal Prescriptions in Onco-Haematology. J. Clin. Pharm. Ther..

[3] Delaloye J., Calandra T. (2014). Invasive Candidiasis as a Cause of Sepsis in the Critically Ill Patient. Virulence.

[4] Hart E., Nguyen M., Allen M., Clark C.M., Jacobs D.M. (2019). A Systematic Review of the Impact of Antifungal Stewardship Interventions in the United States. Ann. Clin. Microbiol. Antimicrob..

[5] Valerio M., Muñoz P., Rodríguez C.G., Caliz B., Padilla B., Fernández-Cruz A., Sánchez-Somolinos M., Gijón P., Peral J., Gayoso J. (2015). Antifungal Stewardship in a Tertiary-Care Institution: A Bedside Intervention. Clin. Microbiol. Infect..

[6] McCormick T.S., Ghannoum M. (2024). Time to Think Antifungal Resistance: Increased Antifungal Resistance Exacerbates the Burden of Fungal Infections Including Resistant Dermatomycoses. Pathog. Immun..

[7] Siopi M., Tarpatzi A., Kalogeropoulou E., Damianidou S., Vasilakopoulou A., Vourli S., Pournaras S., Meletiadis J. (2020). Epidemiological Trends of Fungemia in Greece with a Focus on Candidemia during the Recent Financial Crisis: A 10-Year Survey in a Tertiary Care Academic Hospital and Review of Literature. Antimicrob. Agents Chemother..

[8] Mamali V., Siopi M., Charpantidis S., Samonis G., Tsakris A., Vrioni G., on behalf of the Candi-Candi Network (2022). Increasing Incidence and Shifting Epidemiology of Candidemia in Greece: Results from the First Nationwide 10-Year Survey. J. Fungi.

[9] Sipsas N.V., Pagoni M.N., Kofteridis D.P., Meletiadis J., Vrioni G., Papaioannou M., Antoniadou A., Petrikkos G., Samonis G. (2018). Management of Invasive Fungal Infections in Adult Patients with Hematological Malignancies in Greece during the Financial Crisis: Challenges and Recommendations. Mediterr. J. Hematol. Infect. Dis..

[10] Markogiannakis A., Korantanis K., Gamaletsou M.N., Samarkos M., Psichogiou M., Daikos G., Sipsas N.V. (2021). Impact of a Non-Compulsory Antifungal Stewardship Program on Overuse and Misuse of Antifungal Agents in a Tertiary Care Hospital. Int. J. Antimicrob. Agents.

[11] Papadimitriou-Olivgeris M., Andrianaki A.M., Marangos M., Sipsas N., Apostolidi E.A., Maltezos E., Panagopoulos P., Karapiperis D., Arvaniti K., Perdikouri E.I. (2020). Hospital-Wide Antifungal Prescription in Greek Hospitals: A Multicenter Repeated Point-Prevalence Study. Eur. J. Clin. Microbiol. Infect. Dis..

[12] Donnelly J.P., Chen S.C., Kauffman C.A., Steinbach W.J., Baddley J.W., Verweij P.E., Clancy C.J., Wingard J.R., Lockhart S.R., Groll A.H. (2020). Revision and Update of the Consensus Definitions of Invasive Fungal Disease from the European Organization for Research and Treatment of Cancer and the Mycoses Study Group Education and Research Consortium. Clin. Infect. Dis..

[13] Knitsch W., Vincent J.-L., Utzolino S., Francois B., Dinya T., Dimopoulos G., Ozgunes I., Valía J.C., Eggimann P., León C. (2015). A Randomized, Placebo-Controlled Trial of Preemptive Antifungal Therapy for the Prevention of Invasive Candidiasis Following Gastrointestinal Surgery for Intra-Abdominal Infections. Clin. Infect. Dis..

[14] Patterson T.F., Thompson G.R., Denning D.W. (2016). Practice Guidelines for the Diagnosis and Management of Aspergillosis: 2016 Update by the Infectious Diseases Society of America. Clin. Infect. Dis..

[15] Valerio M., Vena A., Bouza E., Reiter N., Viale P., Hochreiter M., Giannella M., Muñoz P. (2015). COMIC Study Group. How Much European Prescribing Physicians Know about Invasive Fungal Infections Management?. BMC Infect. Dis..

[16] Riccò M., Ranzieri S., Balzarini F., Vezzosi L., Marchesi F., Valente M., Peruzzi S. (2021). A Pilot Study on Knowledge, Attitudes and Beliefs of Medical Professionals on Invasive Fungal Infections (Italy, 2020). J. Med. Mycol..

[17] Oladele R., Otu A.A., Olubamwo O., Makanjuola O.B., Ochang E.A., Ejembi J., Irurhe N., Ajanaku I., Ekundayo H.A., Olayinka A. (2020). Evaluation of Knowledge and Awareness of Invasive Fungal Infections amongst Resident Doctors in Nigeria. Pan Afr. Med. J..

[18] Gómez-Chaves C.I., Díaz-Brochero C., Nocua-Báez L.C., Cortés J.A. (2025). FungiCAP Survey: Insights into Physicians’ Knowledge, Attitudes and Practices in Antifungal Prescriptions in Colombia. J. Eval. Clin. Pract..

[19] Ibrahim S.M., Adlan N., Alomair S.M., Butaiban I., Alsalman A., Bawazeer A., Alqahtani M., Mohamed D., Emeka P.M. (2023). Evaluation of Systemic Antifungal Prescribing Knowledge and Practice in the Critical Care Setting among ICU Physicians and Clinical Pharmacists: A Cross-Sectional Study. Antibiotics.

