# Supplementary material for: Knowledge, Attitudes and Practices of Physicians Regarding Antifungal Therapy in Tertiary Care Patients: A Cross-Sectional Survey in Greece
Source: Pathogens. 2026 Jan 26;15(2):138. doi: 10.3390/pathogens15020138 (PMC12942650; doi:10.3390/pathogens15020138)
Supplement: Supplementary file 1 [file pathogens-15-00138-s001.zip › Questionnaire (1) (1).pdf]

# ***Questionnaire on Antifungal Medications use in Universal Hospital of Heraklion***

Specialty:

○ Internal Medicine

○ Intensive Care

○ Hematology

○ Oncology

○ Surgery

○Other (please specify): __________

Years of Clinical Practice:

○<5 years

○ 5–10 years

○ 11–20 years

○ 20 years

Current Position:

○ Resident

○ Attending Physician

○ Consultant

○ Other (please specify): __________

1. Which of the following antifungal agents is most appropriate for initial treatment of invasive candidiasis in a hemodynamically unstable patient?

○ Fluconazole

○ Micafungin

○ Voriconazole

○ Amphotericin B

○ I am not familiar

2. What is the recommended duration of therapy for uncomplicated candidemia after blood cultures have cleared?

○ 7 days

○ 14 days

○ 21 days

○ 28 days

○ I am not familiar

3. Which antifungal agent requires therapeutic drug monitoring due to variable pharmacokinetics?

○ Fluconazole

○ Micafungin

○ Voriconazole

○ Anidulafungin
○ I am not familiar

4. First choice for invasive aspergillosis:
○ Isavuconazole
○ Posaconazole
○ Voriconazole
○ Fluconazole
○ I am not familiar

5. Which of the following factors does NOT necessitate adjustment of antifungal dosing?
○ Renal impairment
○ Hepatic impairment
○ Patient's age
○ Concomitant use of rifampin
○ I am not familiar

6. Which diagnostic test is most sensitive for early detection of invasive aspergillosis?
○ Serum galactomannan assay
○ Blood cultures
○ Chest X-ray
○ Beta-D-glucan assay
○ I am not familiar

7. The Candida score is a clinical prediction tool used to identify ICU patients at increased risk for invasive candidiasis. Which of the following components is NOT part of the Candida score?
○ Surgery upon ICU admission
○ Total parenteral nutrition
○ Severe sepsis
○ Neutropenia
○ I am not familiar with the score

8. The Candida colonization index is used to assess the risk of invasive candidiasis in ICU patients. What is the threshold value above which the risk of invasive candidiasis is considered significantly increased?
○ 0.2
○ 0.3
○ 0.5
○ 1.0
○ I am not familiar

9. Patients with GI rupture that require prophylactic antifungal treatment:
○ Oesophagus rupture
○ Stomach rupture
○ Small bowel rupture
○ Colon rupture
○ I am not familiar

10. In neutropenic fever, antifungal medication is indicated:
○ First day empirical treatment with broad spectrum antibiotics
○ Part of the empirical antimicrobial scheme if the patient is in septic shock
○ After 48h of empirical antibiotics if the patient is still febrile
○ Only if a culture is positive for fungi
○ I am not familiar

11. Risk factors for invasive fungal infections:
○ Recent broad spectrum antibiotic use
○ Diabetes mellitus
○ Prolonfed and profound neutropenia
○ Chronic kidney disease
○ Hematologic malignancy
○ Allo-HSCT
○ Immunosuppression
○ Total parenteric nutrition
○ CKD
○ Abdominal surgery
○ Central venous catheter
○ Multifocal candida colonisation
○ Corticosteroid use

12. Antifungal medications prescribing by your department is mostly based on:
○ Local guidelines
○ Stanford guide
○ Infectious diseases expert
○ Therapies protocol
○ Artificial intelligence

13. How confident are you in interpreting antifungal susceptibility reports?
○ Very confident
○ Somewhat confident
○ Not confident

14. How often do you consult an ID expert to treat a patient with suspected/confirmed IFI?
○ Always

○ Often
○ Sometimes
○ Rarely
○ Never

15. Do you routinely review antifungal therapy for de-escalation opportunities?
○ Yes
○ No

16. How often do you prescribe antifungal medications?
○ Often
○ Sometimes
○ Rarely
○ Never

17. How familiar are you with the prophylactic use of antifungal medication?
○ Very familiar
○ Somewhat familiar
○ Not familiar

18. How familiar are you with the empirical use of antifungal medication?
○ Very familiar
○ Somewhat familiar
○ Not familiar

19. How familiar are you with the pre-emptive use of antifungal medication?
○ Very familiar
○ Somewhat familiar
○ Not familiar

20. How familiar are you with the targeted use of antifungal medication?
○ Very familiar
○ Somewhat familiar
○ Not familiar

21. How familiar are you with the use of Galactomannan and B-D glucan in clinical practice?
○ Very familiar
○ Somewhat familiar
○ Not familiar

22. The primary goal of an AFS program is to:
○ Reduce overall antifungal consumption
○ Improve clinical outcomes and reduce fungal resistance
○ Prevent healthcare-associated infections

○ Minimize healthcare costs exclusively
○I am not familiar

23. What is the recommended initial treatment strategy for patients with suspected invasive candidiasis in the ICU under an AFS approach?
○ Immediate use of fluconazole in all patients
○ Diagnostic-based treatment supported by biomarkers and imaging
○ Prophylactic antifungal use for all high-risk patients
○ Delay treatment until cultures confirm infection
○ I am not familiar

24. In your clinical practice, how often do you reassess antifungal prescriptions for de-escalation or discontinuation?
○ Always
○ Often
○ Sometimes
○ Rarely
○ Never

25. Do you feel confident in your ability to prescribe antifungal medications appropriately in accordance with AFS principles?
○ Very confident
○ Somewhat confident
○ Not confident

26. Which area of IFI/antifungal use would you benefit from additional training in?
○ Diagnostic tools for fungal infections
○ Principles of antifungal de-escalation
○ Management of antifungal resistance
○ Therapeutic drug monitoring for antifungals
○ Drug-drug interactions involving antifungal medications

27. What is your preferred method for receiving AFS-related education and training?
○ Interactive workshops
○ Online learning modules
○ Case-based discussions
○ Printed guidelines or resource
